# The Alteration of Salivary Immunoglobulin A in Autism Spectrum Disorders

**DOI:** 10.3389/fpsyt.2021.669193

**Published:** 2021-05-21

**Authors:** Wuyi Gong, Yanan Qiao, Bosheng Li, Xiaoguo Zheng, Ruihuan Xu, Mingbang Wang, Xiaohui Mi, Yongming Li

**Affiliations:** ^1^Department of Orthodontics, Shanghai Engineering Research Center of Tooth Restoration and Regeneration, School and Hospital of Stomatology, Tongji University, Shanghai, China; ^2^Shanghai Key Laboratory of Embryo Original Disease, School of Medicine, International Peace Maternity and Child Health Hospital, Shanghai Jiao Tong University, Shanghai, China; ^3^Clinic Lab, Longgang District People's Hospital of Shenzhen, Shenzhen, China; ^4^Shanghai Key Laboratory of Birth Defects, Division of Neonatology, National Center for Children's Health, Xiamen Branch of Children's Hospital of Fudan University (Xiamen Children's Hospital), Children's Hospital of Fudan University, Shanghai, China

**Keywords:** autism spectrum disorders, immunoglobulin A, behavior, *Streptococcus mutans*, polymeric immunoglobulin receptor, mucosal immunity, saliva

## Abstract

**Objectives:** Autism spectrum disorders (ASD) are neurodevelopmental disorders with changes in the gut and oral microbiota. Based on the intimate relationship between the oral microbiota and oral mucosal immunity, this study aimed to investigate changes in salivary immunoglobulin A (IgA) level in ASD and the underlying mechanism for any such changes.

**Methods:** We recruited 36 children diagnosed with ASD and 35 normally developing children and measured their salivary IgA content using enzyme-linked immunosorbent assay (ELISA). The valproate (VPA) -treated ASD mouse model was established by prenatal exposure to valproate and mouse salivary IgA content was also quantified by ELISA. The submandibular glands of VPA and control mice were isolated and analyzed using qRT-PCR, immunofluorescence staining, and flow cytometry. ASD-related *Streptococci* were co-incubated with the human salivary gland (HSG) cell line, and western blotting was used to detect the levels of relevant proteins.

**Results:** We found that salivary IgA content was significantly decreased in patients with ASD and had a significant ASD diagnostic value. The salivary IgA content also decreased in VPA mice and was significantly correlated with autistic-like behaviors among them. The mRNA and protein levels of the polymeric immunoglobulin receptor (*Pigr*) were downregulated in the submandibular glands of VPA mice and the *Pigr* mRNA level was positively correlated with mouse salivary IgA content. HSG cells treated with ASD-related *Streptococci* had reduced PIGR protein level.

**Conclusion:** Therefore, protective IgA levels were reduced in the saliva of individuals with ASD, which correlated with the bacteria-induced downregulation of *Pigr* in salivary glands. This study suggests a new direction for ASD diagnosis and prevention of oral diseases in ASD cohorts and provides evidence for the ASD mucosal immunophenotype in the oral cavity.

## Introduction

Autism spectrum disorders (ASD) are early-onset neurodevelopmental disorders characterized by core deficits in language and social interaction, anxiety, and stereotypic behaviors (1). ASD affect one in 59 children in the United States of America (2), and the prevalence rate in Chinese children has reached 0.70% (3). Despite this high prevalence, the pathogenesis of ASD remains unclear. The role of microbial dysbiosis in ASD etiology is of increasing interest (4). Changes in the gut microbiota have been widely reported in patients with ASD and in ASD mouse models, and accumulating evidence links changes in the gut microbiota to the pathogenesis of ASD (1, 5, 6).

The oral cavity serves as the sole entry point to the gut (7) and recent evidence has indicated a perturbation of the oral microbiota in the population with ASD (8, 9). Compared to normally developed children, the abundance of *Haemophilus* and *Streptococci* has been reported to be significantly higher in children with ASD, whereas the abundance of *Prevotella, Selenomonas, Actinomyces, Porphyromonas*, and *Fusobacterium* has been found to be reduced (9). This altered composition of the oral microbiota is believed to affect oral mucosal immunity, since a delicate balance is maintained by oral mucosal immunity tolerating commensal bacteria while expelling pathogenic antigens. Therefore, salivary immunoglobulin A (IgA) plays an important role (10, 11).

**Figure 1:**
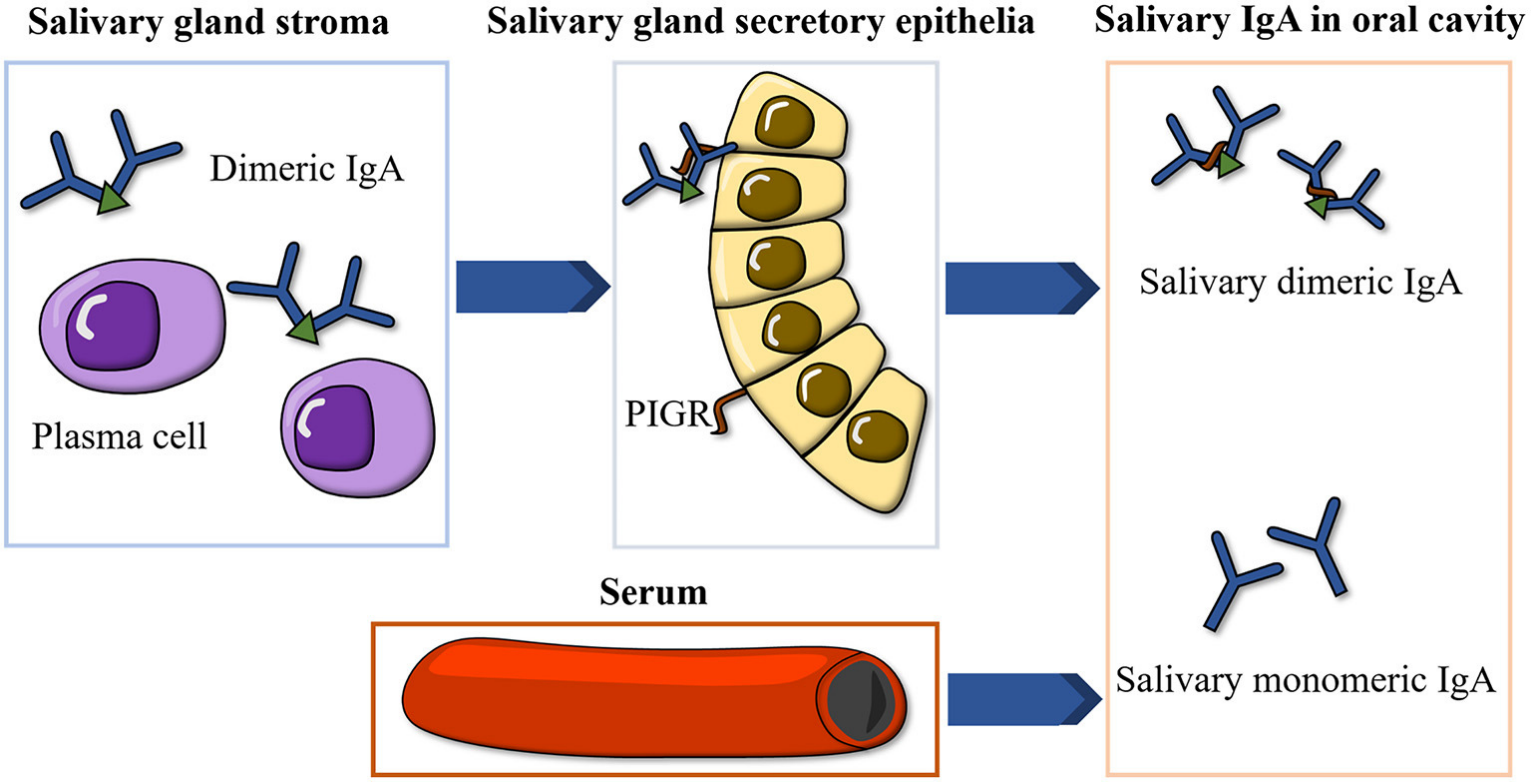
Illustration of the formation of salivary IgA. IgA, immunoglobulin A; PIGR, polymeric immunoglobulin receptor.

IgA is the most abundant immunoglobulin isotype in saliva and occurs in two forms: dimeric IgA, produced by local plasma cells in the stroma of salivary glands and transported to the oral cavity by the polymeric immunoglobulin receptor (PIGR) on salivary gland ductal cells, and monomeric IgA, derived from serum (12) (Figure 1). Salivary IgA is the major protective antibody in oral mucosal immunity, acting in concert with the innate immune system to inhibit microbial adhesion to mucosal and dental surfaces, promoting the elimination of cariogenic microorganisms such as *Streptococcus mutans* (13, 14). Salivary IgA deficiency predisposes patients to oral mucosal infections and dental caries (15, 16).

Worse oral health in ASD cohorts has been observed in previous studies when compared to individuals with no ASD (9, 17). In light of the altered oral health status in patients with ASD, and the correlations between oral microbiota dysbiosis and mucosal immunity, we posit that the level of salivary IgA, which is possibly affected by oral bacteria, may shift in subjects with ASD. This study measured salivary IgA levels in children with ASD and an ASD mouse model and explored the cause of salivary IgA alteration through *in vivo* and *in vitro* experiments. Existing evidence of abnormal ASD mucosal immunity is mostly centralized in the gastrointestinal tract, whereas that in the oral cavity remains unexplored. This study aims to fill this gap to improve the diagnosis and oral health care among individuals with ASD and gain a better understanding of the ASD mucosal immunophenotype in the oral cavity.

## Materials and Methods

### Ethics Statement

The procedures of both human and animal experiments were approved by the Ethics Committee of the School and Hospital of Stomatology, Tongji University (SL2019SR19 and SL2019DW42), and were conducted in strict accordance with relevant guidelines and regulations. Written informed consent was obtained prior to the study from all participants or their guardians.

### Study Participants and Sample Collection

ASD children diagnosed according to the Diagnostic and Statistical Manual of Mental Disorders, 5th Edition (DSM-5) were recruited locally in the Affiliated Stomatology Hospital of Tongji University. Thirty-six ASD children (31 males and 5 females) between 4 and 14 years of age were enrolled. They had no other systematic disease or neurodevelopmental disorder, no long-term drug therapy history, no fever, influenza history or antibiotic/antifungal use within 1 month, and no oropharynx inflammatory conditions. Thirty-five gender- and age-matched typically developing (TD) children between 6 and 14 years of age were also recruited. The saliva collection was performed as previously described (9). Between 14:00 and 17:00, ~1 ml of non-stimulated, natural outflow saliva was collected and transferred into 1.5 ml sterile Eppendorf tubes. All samples were immediately placed on ice, transported to the laboratory within 2 h, and stored at −80°C until salivary IgA measurement. In total, 71 saliva specimens from 36 ASD and 35 TD children were obtained.

### Valproate Acid-treated ASD Mouse Model

**Figure 2:**
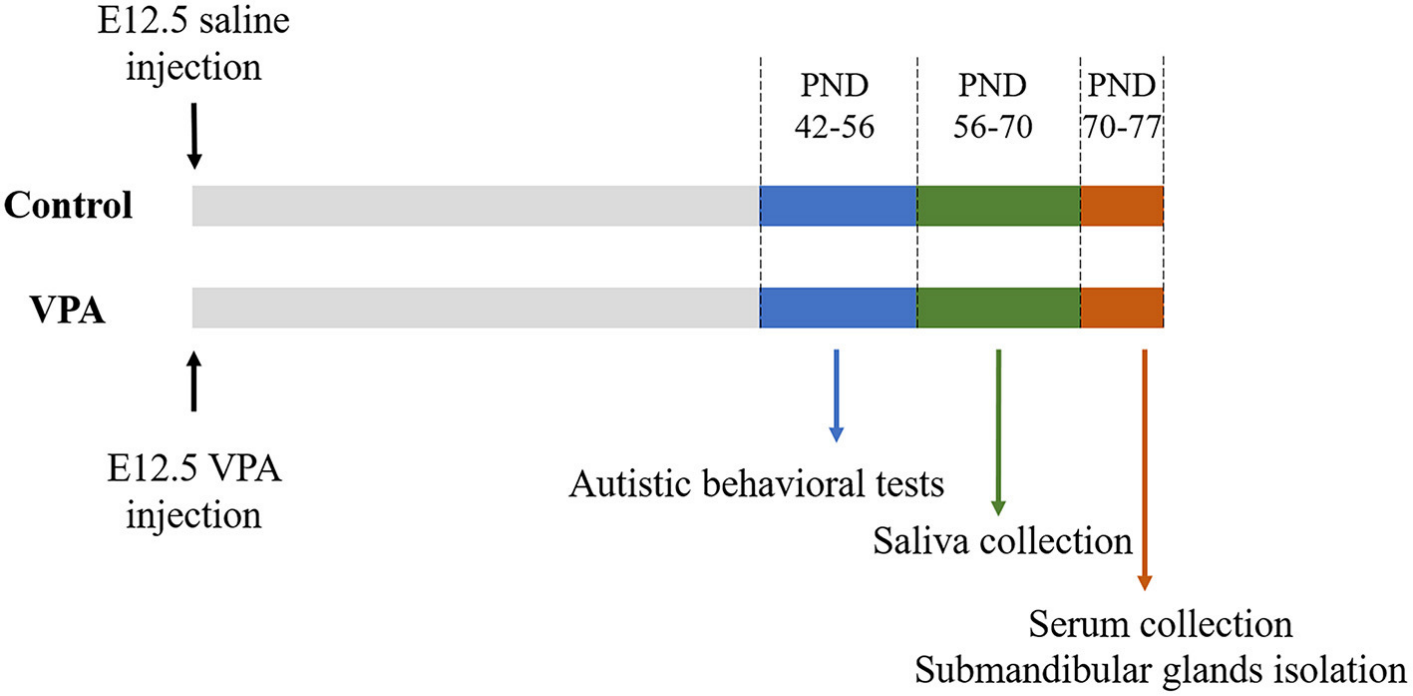
The outline of animal experiments. VPA, valproate acid; E, embryonic day; PND, postnatal day.

C57BL/6 mice were housed in the animal facility of Tongji University, Shanghai, China under specific pathogen-free conditions at 22 ± 2°C under a 12:12 h light: dark cycle, with food and water provided *ad libitum*. Male and female mice were housed separately in groups of five mice each. Timed pregnant mice at embryonic day (E) 12.5 received a single intraperitoneal injection (500 mg/kg) of sodium valproate (Sigma-Aldrich, St. Louis, MO, USA) dissolved in 0.9% saline or an equal volume of 0.9% saline. Females were housed individually and allowed to raise their own litters. The offspring were weaned on postnatal day (PND) 21. The male offspring of the VPA-injected mothers were considered as the ASD mouse model, whereas that of the saline-injected mothers was used as the control group (18). All subsequent experiments were performed only on male offspring during PND 42–77. The timeline of the animal experiments is shown in Figure 2.

### Autistic Behavioral Tests

Before performing the autism behavioral tests, the animals were acclimatized to the experimental room for 1 day. An interval of at least 1 d was ensured between the different tests, and all tests were conducted between 14:00 and19:00 h. The apparatus was cleaned with 75% ethanol between the trials.

#### Three-chamber Social Test

PND 42–56 mice were introduced into a three-chamber social interaction apparatus (plastic, 60 × 40 × 20 cm). Openings between the compartments allowed the animals to access all three chambers. In the first phase, the test mouse was allowed to freely explore the environment for 10 min for habituation. In the second phase, the mouse was gently guided to the central chamber, and the two entrances were blocked. Two small containers that were either empty or contained an age- and sex-matched mouse (Stranger 1) were placed in the left and right chambers, respectively. Then, the two entrances were opened to allow the test mouse to freely explore the new environment for 10 min. In the third phase, the test mouse was gently guided to the center chamber again, and the entrances were blocked. Another age- and sex-matched mouse (Stranger 2) was placed into the empty container, and the test mouse was then allowed to explore Stranger 1 and 2 for another 10 min. The time the test mouse spent in each chamber and interacting with each container in phase 2 and phase 3 was recorded. The Sociability and social preference indices were calculated using the following equations (19, 20).

$$\begin{array}{l} \text{Sociability index}=\frac{\text{Time spent in Stranger }1\text{ chamber}\left(\text{phase}2\right)}{\text{Time spent in empty chamber}\left(\text{phase}2\right)}\\ \text{Social preference index}\\ =\frac{\text{Time spent in Stranger }2\text{ chamber}\left(\text{phase}3\right)}{\text{Time spent in Stranger }1\text{ chamber}\left(\text{phase}3\right)} \end{array}$$

#### Self-grooming Test

A self-grooming test was conducted during the first phase of the three-chamber social test. The self-grooming behavior of the test mice was recorded for 10 min.

#### Open Field Test

PND 42–56 mice were placed in the open field instrument (Med Associates Inc., St Albans, VT, USA, 40 × 40 × 20 cm) and allowed to explore freely for 10 min while continually monitoring their position using the Activity Monitor 7 software (Med Associates Inc.). The time spent in the center zone, defined as the interior 15 × 15 cm, was recorded.

#### Light-dark Test

Individual PND 42–56 mice were placed in the dark compartment of a light-dark test apparatus (plastic, 50 × 30 × 30 cm). Openings between the light and dark compartments provided free access to the compartments. The time that the mice spent in the light compartment was recorded in a 10-min period.

### Animal Sample Collection

PND 56–70 mice were anesthetized by an intraperitoneal injection of 2% pentobarbital sodium. Saliva was stimulated by an intraperitoneal injection of 500 μg/kg sterile pilocarpine (Sigma-Aldrich) and harvested using capillary pipets. Blood was collected from the orbits of anesthetized PND 70–77 mice, and the serum was separated after centrifugation. The mice were then euthanized, and the submandibular glands were removed for subsequent experiments. Saliva and serum samples were collected between 14:00 and 17:00 h and stored at −80°C until analysis.

### Salivary and Serum IgA Measurement

Human and mouse IgA levels were measured in human and mouse saliva samples, respectively, using enzyme-linked immunosorbent assay (ELISA) kits (Multiscience, Hangzhou, China), according to the manufacturer's instructions.

### Quantitative Real-time Polymerase Chain Reaction

Total RNA of isolated mouse submandibular glands was extracted using TRIzol® reagent (Takara Ltd., Otsu, Japan) and cDNA was synthesized using PrimeScript RT Master Mix (Takara Ltd.). qRT-PCR was conducted using qPCR SYBR Green Master Mix (Yeasen, Shanghai, China) and a LightCycler®96 instrument (Roche, Mannheim, Germany). Glyceraldehyde-3-phosphate dehydrogenase (Gapdh) was analyzed as a housekeeping gene. The primer sequences (Sangon, Shanghai, China) are listed in Supplementary Table 1.

### Flow Cytometry

Mouse submandibular gland tissues were cut into pieces and resuspended in 1,000 μL of RPMI1640 medium (Hyclone, Logan, UT, USA) containing 0.002% deoxyribonuclease I (Sigma-Aldrich), 0.1% type IV collagenase (Sigma-Aldrich) and 0.01% hyaluronidase (Sigma-Aldrich) for 15 min. The single-cell suspension was then incubated with FITC-conjugated rat anti-mouse IgA antibody (BD Pharmingen, San Jose, CA, USA). Data were acquired by flow cytometry (Beckman Coulter, Brea, CA, USA) and analyzed using FlowJo software (FlowJo LLC).

### Cell Culture

The human salivary gland (HSG) cell line was provided by the Shanghai Engineering Research Center of Tooth Restoration and Regeneration (Tongji University, Shanghai, China). HSG cells were incubated with Dulbecco's modified Eagle's medium (Hyclone) containing penicillin, streptomycin (Hyclone), and 10% fetal bovine serum (Gibco, Grand Island, NY, USA) at 37°C in a 5% CO_2_ incubator. The culture solution was replaced every day, and HSG cells were passaged every 2 days. HSG cells were adjusted to ~5 × 10^5^ cells per 6 cm culture plate for bacterial co-incubation.

*Streptococcus mutans* (ATCC 25175) was purchased from ATCC (ATCC, Manassas, VA, USA) and grown to mid-log phase [optical density (OD) = 0.30, according to the growth curve shown in Supplementary Figure 1] in brain heart infusion (BHI) broth (Hopebio, Qingdao, China) at 37°C. The colony-forming units (CFU)/mL of *Streptococcus mutans* at 0.30 OD was estimated (data not shown). Bacteria were then heat-killed in BHI broth at 60°C for 30 min and added to the HSG cells at a ratio of 100 bacteria to 1 cell and co-incubated for 24 h, these were determined as optimal co-incubation conditions by preliminary experiments (data not shown).

### Immunofluorescence Staining

For immunofluorescence staining, mouse submandibular glands were fixed in 4% paraformaldehyde, then sequentially dehydrated in 15 and 30% sucrose solution, and frozen in Tissue-Tek OCT (Sakura, Finetec, Torrance, CA, USA). Ten-micron cryosections were mounted on glass slides, blocked with 0.5% bovine serum albumin (Sangon), and stained using rabbit anti-mouse PIGR antibody (1:200, A6130, Abclonal, Wuhan, China) and Alexa 488-conjugated goat anti-rabbit secondary antibody (1:1,000, A32731, Invitrogen, Carlsbad, CA, USA). HSG cells were cultured in 24-well plates on glass coverslips at a density of 5,000 cells/well for 24 h and fixed with 4% paraformaldehyde. After permeabilization with Triton X-100 (Beyotime, Shanghai, China), the cells were washed with phosphate-buffered saline (PBS) and blocked with 5% bovine serum albumin (Sangon) for 30 min at 20°C. Mouse anti-human cytokeratin 14 antibody (1:1,000, MA5-11599, Invitrogen) and rabbit anti-human PIGR antibody (1:200, A6130, Abclonal) were applied overnight at 4°C. Secondary antibody incubations were carried out for 1 h at room temperature using Alexa 488-conjugated goat anti-rabbit (1:1,000, A32731, Invitrogen) and Alexa 594-conjugated donkey anti-mouse secondary antibodies (1:1,000, A21203, Invitrogen). The coverslips were mounted on glass slides with an embedding medium. All sections were counterstained with DAPI (Sigma–Aldrich) for 5 min. Slides were observed using a fluorescence microscope (Nikon, Tokyo, Japan) and a confocal laser scanning microscope (Nikon).

### Western Blotting

HSG cells were washed with PBS and lysed with RIPA buffer (Beyotime). The cell lysates were separated on 10% SDS-PAGE gels and transferred to polyvinylidene fluoride membranes (Millipore, Bedford, MA, USA). Membranes were blocked with 3% bovine serum albumin (Sangon) in TBS for 2 h and then incubated with primary antibodies against GAPDH (AF1186, Beyotime), PIGR (A6130, Abclonal), NF-κB (8242S, Cell Signaling Technology, Beverly, MA, USA), and phospho-NF-κB (3033, Cell Signaling Technology) at 1:1,000, overnight at 4°C. Primary antibodies were labeled by incubation with secondary antibodies (1:10,000, 5151s, Cell Signaling Technology) at 20°C for 1.5 h. Antibody-bound proteins were detected using an Odyssey® CLx Imaging System (LI-COR, Lincoln, NE, USA). GAPDH antibody levels were used for normalization.

### Statistical Analyses

Normality was first determined by Shapiro-Wilk tests, and the homogeneity of variance was checked by the Brown-Forsythe test. The Student's *t*-test was used if two sets of continuous variables were normally distributed; otherwise, the Mann-Whitney U-test was employed. One-way analysis of variance (ANOVA) was used if three sets of continuous variables were normally distributed and met the condition of variance homogeneity; otherwise, the Kruskal-Wallis test was employed. Categorical data were analyzed using the Chi-squared test. The demographic information of children with ASDs and TD children was compared using the Student's *t*-test (age) and the Chi-squared test (gender). Human salivary IgA content was analyzed using the Student's *t*-test, and the diagnostic performance of human salivary IgA was assessed by receiver operator characteristic (ROC) analysis. The data of mouse behavioral tests and mouse salivary IgA content were analyzed using the Student's *t*-test and the Mann-Whitney U-test. The correlation between mouse salivary IgA and mouse autistic-like behaviors was evaluated using Pearson's correlation analysis. The comparison of qRT-PCR data was performed using the Student's *t*-test and the Mann-Whitney U-test. The correlation between mouse salivary IgA content and the mouse *Pigr* mRNA levels was evaluated using Pearson's correlation analysis. The data from flow cytometry and mouse serum IgA content were analyzed using the Mann-Whitney U-test. The quantification data of immunofluorescence staining were compared using the Student's *t*-test. One-way ANOVA was performed on the quantification data of western blotting. All data are presented as the mean ± standard error of mean (SEM), and *p* < 0.05, which was considered statistically significant. Statistical analyses were performed using the SPSS software (IBM, Armonk, NY, USA). The quantification of immunofluorescence staining and western blotting was performed using ImageJ (National Institutes of Health, Bethesda, MD, USA).

## Results

### Salivary IgA Content Is Reduced in Patients With ASD and Has Diagnostic Value

**Figure 3:**
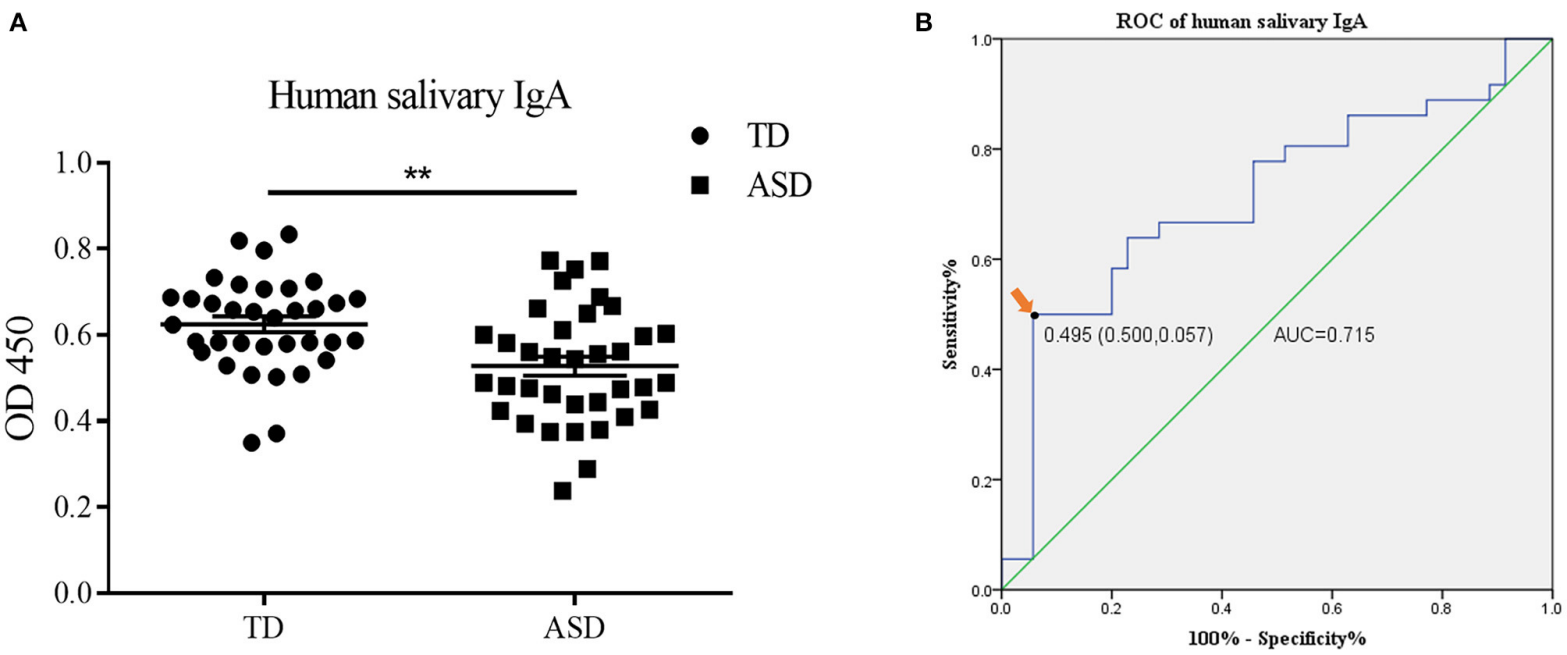
The salivary immunoglobulin A (IgA) content in human saliva samples and the diagnostic value of human salivary IgA in Autism Spectrum Disorders (ASD). **(A)** The IgA content (presented as optical density at 450 nm) in the saliva of the typically developing group and ASD children was determined by enzyme-linked immunosorbent assay. The above data are presented as the mean ± SEM, *n* = 35–36 per group, two replicate experiments, each data point represents results from an individual child, Student's *t*-test, ** denotes *p* < 0.01. **(B)** The receiver operator characteristic (ROC) curve and the area under the ROC curve (AUC) of the salivary IgA in ASD diagnosis. The arrow indicates the optimal cutoff point of salivary IgA.

The demographic information of all participants is presented in Table 1. Thirty-five typically developing (TD) children between 6 and 14 years of age and 36 ASD children between 4 and 14 years of age were enrolled. No statistical difference was observed in the age (9.03 ± 0.351 vs. 9.61 ± 0.442, Student's *t*-test, *p* = 0.308) and gender ratio (27 males and 8 females vs. 31 males and 5 females, Chi-squared test, *p* = 0.329) between two groups. To examine the salivary IgA level in ASD and TD groups, saliva samples were tested by ELISA. The salivary IgA content of patients with ASD was significantly lower than that of the TD group (0.62 ± 0.018 vs. 0.53 ± 0.022, Student's *t*-test, *p* = 0.001) (Figure 3A). We subsequently evaluated the diagnostic performance of salivary IgA and found that the area under the ROC curve (AUC) reached 71.5% (*p* = 0.002), indicating that the salivary IgA level was effective for ASD diagnosis. The cut-off point for salivary IgA was determined to be 0.495 (sensitivity, 0.500; specificity, 0.943), as shown in Figure 3B.

**Table 1:** Demographic information of the subjects.

	**TD (*n* = 35)**	**ASD (*n* = 36)**	***p*-value**
Age (mean ± SEM)^†^ (range)	9.03 ± 0.351 6–14	9.61 ± 0.442 4–14	0.308 \
Gender ratio (M/F)^‡^	27/8	31/5	0.329

*TD, typically developing group; ASD, autism spectrum disorders; SEM, standard error of mean; M/F, male/female*.

† *Calculated using the Student's t-test*.

‡ *Calculated using the Chi-squared test*.

### The Salivary IgA Content Decreases in the VPA-treated ASD Mouse Model and Correlates With Mouse Autistic-like Behaviors

**Figure 4:**
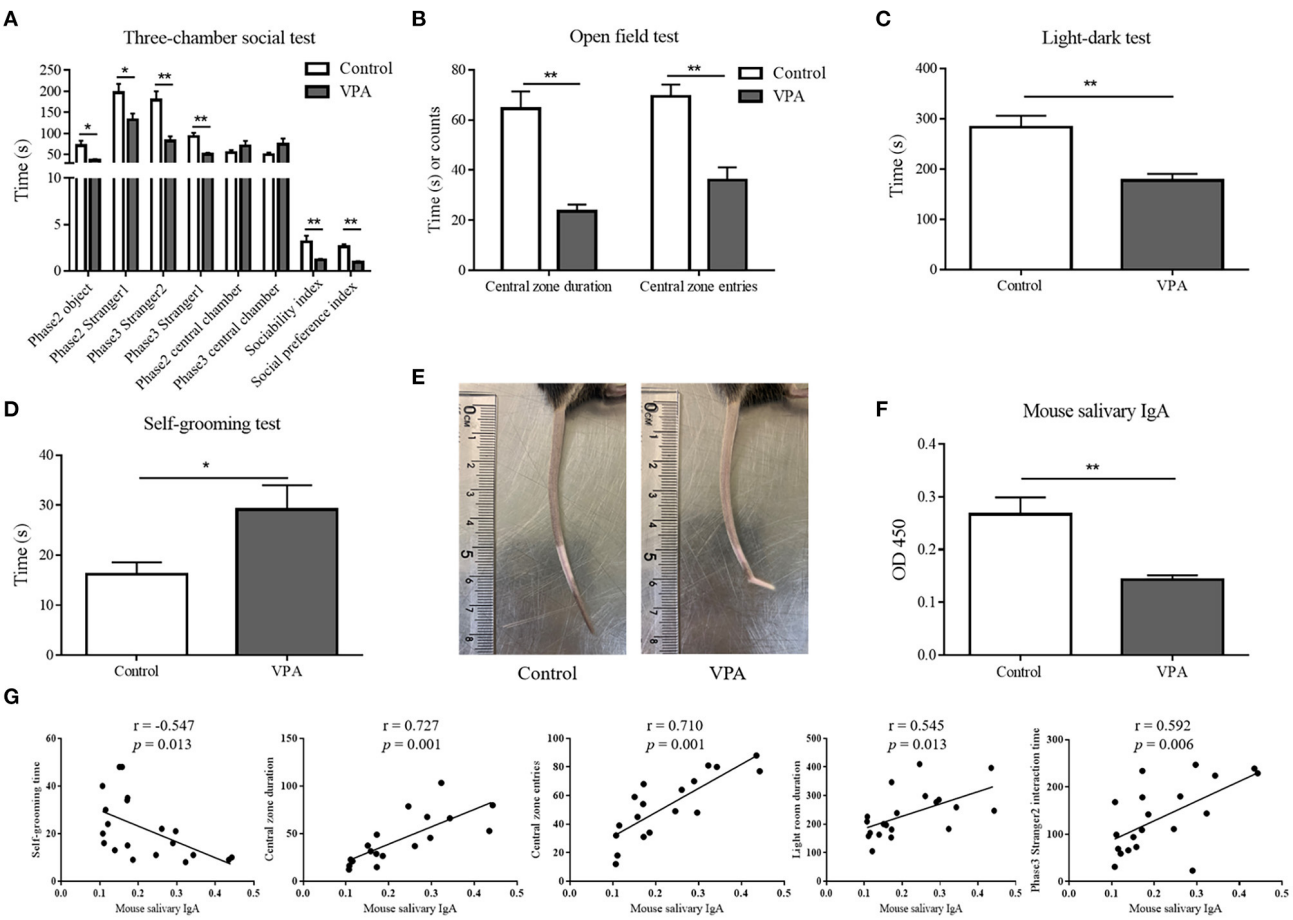
The salivary immunoglobulin A (IgA) content in mouse saliva samples and its correlation with the mouse autistic-like behaviors. **(A)** In the three-chamber social performance test, the durations of valproate acid (VPA) mice and control mice interacted with the object, Stranger 1 and Stranger 2 in phase 2 and phase 3, and the time they spent in each chamber were recorded. According to which the sociability index and social preference index were calculated (*n* = 9–11 per group, Mann-Whitney U-test and Student's *t*-test). **(B)** The time VPA mice and control mice spent in the central zone and the frequency of them entering the central zone in the open field test (*n* = 9–11 per group, Student's *t*-test). **(C)** The time VPA mice and control mice spent in the lightroom in the light-dark test (*n* = 9–11 per group, Student's *t*-test). **(D)** The time VPA mice and control mice spent in self-grooming (*n* = 9–11 per group, Student's *t-*test). **(E)** A representative picture of the “kink” in the tails of VPA mice. **(F)** The IgA content (presented as optical density at 450 nm) of mouse saliva samples was determined by enzyme-linked immunosorbent assay (*n* = 6 per group, two replicate experiments, Student's *t*-test). The above data are presented as the mean ± SEM, * denotes *p* < 0.05, ** denotes *p* < 0.01. **(G)** The Pearson's correlation analysis of salivary IgA content and mouse autistic-like behaviors, including self-grooming time, time spent in the central zone, central zone entries, time spent in the lightroom, and phase 3 Stranger 2 interaction time.

First, to confirm the establishment of the VPA-treated ASD mouse model, VPA mice were assessed using behavioral tests. In the second phase of the three-chamber social test, VPA mice interacted less with both the empty chamber (the object) (71.45 s ± 11.281 vs. 36.22 s ± 3.104, Student's *t*-test, *p* = 0.013) and the mouse named Stranger 1 (197.18 s ± 20.508 vs. 131.89 s ± 15.366, Student's *t*-test, *p* = 0.025). Although VPA mice stayed slightly longer in the central chamber, the difference was not statistically significant (54.36 s ± 6.388 vs. 70.56 s ± 12.045, Student's *t*-test, *p* = 0.227). However, the sociability index of VPA mice was significantly lower than that of control mice (3.14 ± 0.657 vs. 1.18 ± 0.130, Mann-Whitney U-test, *p* < 0.001). In the third phase, there was a noticeable impairment of VPA mice interacting with the mouse named Stranger 2 (179.73 s ± 20.466 vs. 82.44 s ± 10.799, Student's *t*-test, *p* = 0.001), and less time was spent interacting with Stranger 1 (92.36 s ± 9.083 vs. 50.33 s ± 4.183, Student's *t*-test, *p* = 0.001). VPA mice also lingered longer in the central chamber, however, the difference was not statistically significant (49.55 s ± 5.347 vs. 74.56 s ± 13.646, Student's *t*-test, *p* = 0.083). The social preference index of VPA mice was significantly lower than that of control mice (2.59 ± 0.283 vs. 0.944 ± 0.113, Student's *t*-test, *p* < 0.001) (Figure 4A). VPA mice also exhibited anxiety-like behaviors in the open field test, with fewer central zone entries (69.44 ± 4.649 vs. 36.00 ± 5.121, Student's *t*-test, *p* < 0.001) and less time spent in the central zone (64.43 s ± 6.928 vs. 23.49 s ± 2.774, Student's *t*-test, *p* < 0.001). Similarly, in the light-dark test, VPA mice spent less time in the lightroom than control mice (282.72 s ± 23.162 vs. 177 s ± 13.005, Student's *t*-test, *p* = 0.001) (Figures 4B,C). VPA mice spent more time grooming themselves (16.18 s ± 2.408 vs. 29.11 s ± 4.886, Student's *t*-test, *p* = 0.022) and exhibited more stereotypic behaviors than the control mice (Figure 4D). In addition, VPA mice also developed the characteristic tail “kink” (Figure 4E), as previously reported (21). The behavioral tests and physical malformations validated the successful establishment of the VPA-treated mouse model of ASD.

Saliva samples of VPA and control mice were tested by ELISA. The decline in the salivary IgA content observed in individuals with ASD was also observed in VPA mice (0.27 ± 0.032 vs. 0.14 ± 0.008, Student's *t*-test, *p* = 0.001) (Figure 4F). Pearson's correlation analysis indicated that the mouse salivary IgA content was negatively correlated with self-grooming time (*r* = −0.547, *p* = 0.013), and positively correlated with the time spent in the central zone (*r* = 0.727, *p* = 0.001), central zone entries (*r* = 0.710, *p* = 0.001), time spent in the lightroom (*r* = 0.545, *p* = 0.013) and phase 3 Stranger 2 interaction time (*r* = 0.592, *p* = 0.006) (Figure 4G).

### Downregulation of *Pigr* Levels in the Submandibular Glands of VPA Mice Is Positively Correlated With the Reduction in Salivary IgA

To explore the reason for the decreased salivary IgA content in VPA mice, the origin of salivary IgA needed to be clarified. It is known that salivary IgA consists of monomeric IgA derived from serum and dimeric IgA produced by local plasma cells in the stroma of salivary glands and transported into the oral cavity by PIGR on salivary gland ductal cells.

**Figure 5:**
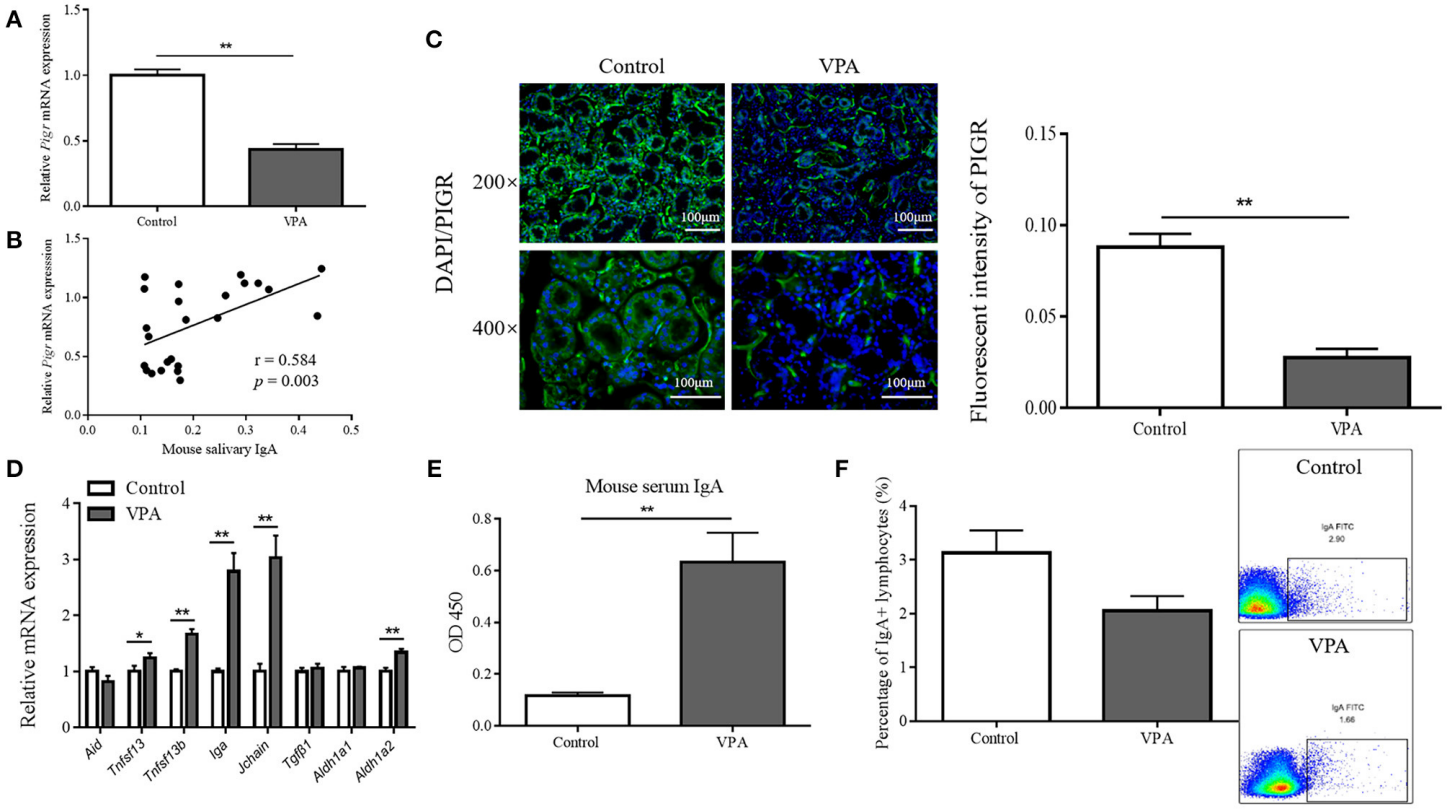
The polymeric immunoglobulin receptor (*Pigr*) levels in mouse submandibular glands and the correlation of which with the mouse salivary immunoglobulin A (IgA) content. **(A)** The qRT-PCR result of the relative mRNA level of *Pigr* in the submandibular glands of valproate acid (VPA) mice and control mice (*n* = 5 per group, Mann-Whitney U-test). **(B)** The Pearson's correlation analysis of the relative *Pigr* mRNA level and mouse salivary IgA content. **(C)** The PIGR protein level in the submandibular glands of VPA mice and control mice confirming by immunofluorescence staining and the quantification results (measuring with fluorescence intensity at 400× magnification, *n* = 5 per group, Student's *t*-test). Representative images of similar results are shown. **(D)** The qRT-PCR results of the relative mRNA levels of genes regulating IgA class-switch recombination, IgA synthesis, and the IgA response in the submandibular glands of VPA mice and control mice (*n* = 5 per group, Mann-Whitney U-test and Student's *t*-test). **(E)** The IgA content (presented as optical density at 450 nm) of mouse serum samples was measured by enzyme-linked immunosorbent assay (*n* = 5–6 per group, two replicate experiments, Mann-Whitney U-test). **(F)** Flow cytometry results showed the percentage of IgA-producing lymphocytes in the submandibular glands of VPA mice and control mice (*n* = 5–6 per group, Mann-Whitney U-test). The above data are presented as the mean ± SEM, * denotes *p* < 0.05, ** denotes *p* < 0.01.

Therefore, we first assessed the *Pigr* levels in mouse submandibular glands. Consistent with the reduction in salivary IgA, the messenger RNA (mRNA) and protein levels of *Pigr* in the submandibular glands of VPA mice were found to be significantly downregulated by qRT-PCR (0.071 ± 0.003 vs. 0.031 ± 0.003, Mann-Whitney U-test, *p* < 0.001) and immunofluorescence staining (0.088 ± 0.007 vs. 0.028 ± 0.005, Student's *t*-test, *p* = 0.0079), respectively (Figures 5A,C). Furthermore, there was a significant positive correlation between mouse salivary IgA content and *Pigr* mRNA level (Pearson's correlation analysis, *r* = 0.584, *p* = 0.003) (Figure 5B).

Next, we tested for changes in the factors that regulate salivary IgA in VPA mice. B cells in the salivary gland stroma undergo IgA class-switch recombination to produce IgA (22). This process is facilitated by activation-induced cytidine deaminase (AID) (23) and two members of the tumor necrosis factor family of ligands, TNFSF13 and TNFSF13b (24). The mRNA level of *Aid* was not affected in the submandibular glands of VPA mice, while the levels of *Tnfsf* 13 (Mann-Whitney U-test, *p* = 0.0444) and *Tnfsf* 13b (Student's *t*-test, *p* < 0.001) were upregulated (Figure 5D). Dimeric IgA is synthesized by plasma cells that link one J-chain molecule to two IgA monomers (25), and the expression of *Iga* (Mann-Whitney U-test, *p* < 0.001) and *Jchain* (Mann-Whitney U-test, *p* < 0.001) were elevated at the mRNA level in VPA mice (Figure 5D). Transforming growth factor-β1 (TGFβ1) (26) together with retinaldehyde dehydrogenase 1 (ALDH1a1) and ALDH1a2, which are important enzymes in the synthesis of retinoic acid (RA), cooperate to promote the overall IgA response (27). However, no significant change was found in the mRNA levels of *Aldh1a1* and *Tgf*β*1* in VPA mice, while *Aldh1a2* expression was elevated (Student's *t*-test, *p* < 0.001) (Figure 5D). In summary, we found no alterations in any of the tested salivary IgA-regulating factors that could cause a decrease in salivary IgA level in VPA mice.

To determine whether serum-derived monomeric IgA or the number of IgA-producing lymphocytes contributed to decreased salivary IgA level, we measured the serum IgA content and the percentage of IgA-producing lymphocytes. The serum IgA content of VPA mice was significantly elevated (0.12 ± 0.012 vs. 0.63 ± 0.113, Mann-Whitney U-test, *p* < 0.001) (Figure 5E), and flow cytometry showed no significant difference between the percentage of IgA-producing lymphocytes (3.13% ± 0.423 vs. 2.06% ± 0.273, Mann-Whitney U-test, *p* = 0.247) within the submandibular glands of VPA and control mice (Figure 5F). This indicated that neither could account for the decreased salivary IgA content in the VPA mice.

### ASD-related *Streptococci* Downregulate the PIGR Protein Level in HSG Cells Which Involves the NF-κB Signaling Pathway

As previously stated, the composition of the oral microbiota is altered in patients with ASD compared with normal individuals; for example, the abundance of *Streptococci* is significantly increased in patients with ASD (9). Given that bacterial signals can modulate *Pigr* (28), an increased amount of *Streptococci* may contribute to the downregulation of *Pigr* in the submandibular glands. *Streptococcus mutans* was selected from the ASD-related *Streptococci* for subsequent experiments because of its high abundance in the oral cavity and its negative correlation with salivary IgA (14).

**Figure 6:**
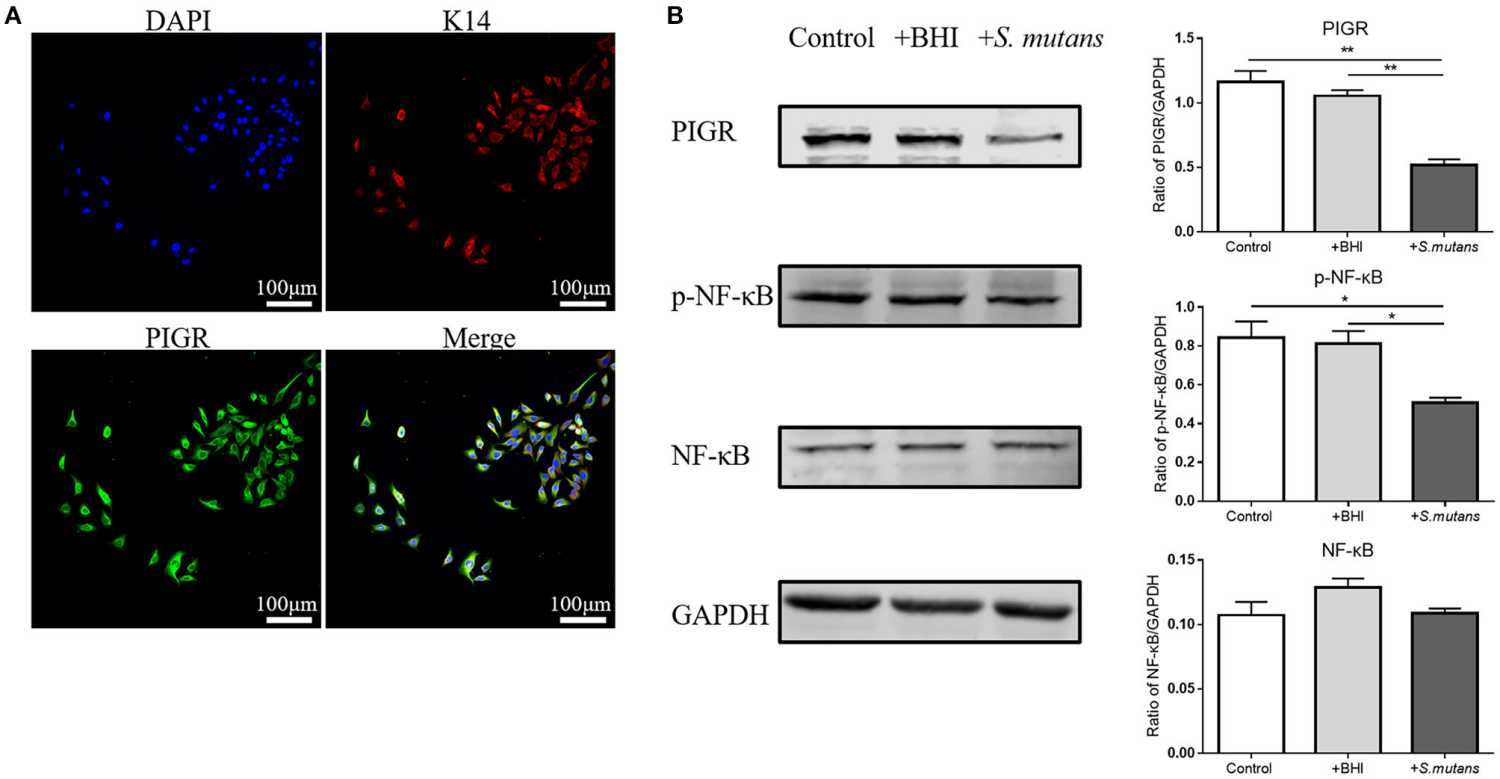
The effect of Autism spectrum disorders (ASD) -related *Streptococci* on the polymeric immunoglobulin receptor (PIGR) protein level and the NF-κB signaling pathway in the human salivary gland (HSG) cells. **(A)** Cytokeratin 14 and PIGR expression in HSG cells was validated by immunofluorescent staining. Representative images of similar results are shown. **(B)** Control group: normal HSG cells; +BHI group: HSG cells treated with blank brain heart infusion broth; +*S. mutans* group: HSG cells co-incubated with heat-killed *Streptococcus mutans*. Western blotting was used to determine the protein levels of PIGR, NF-κB, and phosphorylated NF-κB (p-NF-κB). Representative pictures of western blots with similar results and the quantification of protein levels are shown. The above data are presented as the mean ± SEM, *n* = 3 per group, One-way ANOVA, * denotes *p* < 0.05 ** denotes *p* < 0.01.

First, the expression of *Pigr* and cytokeratin 14 [a marker of salivary ducts (29)] in HSG cells was validated by immunofluorescence staining (Figure 6A). Next, heat-killed *Streptococcus mutans* in BHI broth were added to HSG cells for co-incubation. HSG cells alone and HSG cells treated with blank bacteria medium (BHI broth) served as the control and BHI-treated groups, respectively. The representative western blots and the quantification results of western blotting indicated an inhibitory effect of the bacteria on the protein level of PIGR and NF-κB phosphorylation in HSG cells (Figure 6B). The PIGR protein level was significantly downregulated in the bacteria-treated group compared with the control (1.16 ± 0.086 vs. 0.52 ± 0.045, One-way ANOVA, *p* = 0.001) and the BHI-treated (1.06 ± 0.044 vs. 0.52 ± 0.045, One-way ANOVA, *p* = 0.002) groups. NF-κB phosphorylation was concurrently inhibited in the bacteria-treated group compared with both the control (0.84 ± 0.084 vs. 0.51 ± 0.025, One-way ANOVA, *p* = 0.023) and the BHI-treated (0.78 ± 0.097 vs. 0.51 ± 0.025, One-way ANOVA, *p* = 0.036) groups, while the NF-κB protein level remained unchanged among the three groups.

## Discussion

Mounting evidence supports that neurodevelopmental abnormalities in rodents exposed to valproate *in utero* bear striking similarities to behavioral, cellular, and molecular alterations observed in patients with ASD (30–32). Liu et al. reported that the VPA rodent model could also mimic the microbiome features of autism (33). Therefore, the VPA-treated rodent model can be considered as one of the best-suited animal models for ASD studies. In this work, the VPA-treated mouse model was established, and three-chamber social, self-grooming, open field, and light-dark tests were used to evaluate the social, stereotypic, and anxiety-like behaviors of VPA mice. In the three-chamber social test, VPA mice were found to interact less with both the object and stranger mice, which displayed more anxiety than control mice. Although VPA mice spent a slightly longer time in the central chamber, the difference was not statistically significant, while another study reported a significant increase of central chamber durations in VPA rats (31). This difference may be due to the fewer number of mice we used in our study. However, the decreased sociability and social preference indices observed in VPA mice, compared to control mice, suggested an impairment of their sociability and social preference, similar to previous observations (19, 20). Moreover, the results from the self-grooming, open field, and light-dark tests showed that VPA mice exhibited more anxiety-like and stereotypic behaviors than control mice, which was consistent with previous studies (19, 31, 34). The autistic-like behaviors of VPA mice indicated the successful establishment of the VPA-treated ASD mouse model.

We found that the salivary IgA content was significantly lower in both children with ASD and VPA mice than in the controls. A previous study in Venezuela recruited 34 children with ASD and 34 healthy controls aged 4–13 and reported a declining tendency in salivary IgA content among children with ASD, with no statistical significance. This may stem from the data deviation caused by the circadian rhythmicity of human salivary IgA content (35), since the author did not state whether the saliva collections were all conducted at a unified time point. Salivary IgA is the primary protective antibody in the oral cavity. It inhibits bacterial adherence to epithelial and tooth surfaces, reduces the accumulation of human dental plaque by targeting glucosyl-transferases of *Streptococci*, and cooperates with innate immune factors in saliva (36). Therefore, reduced IgA levels may affect the oral health status. A higher decayed-missing-filled teeth/surface index, gingival index, bleeding on probing index, more prevalent halitosis, and more frequent oral lesions in ASD cohorts reflect the severity of dental caries and poor dental hygiene associated with ASD (7, 9, 17). The high prevalence of caries and poor oral health have been formerly ascribed to impaired manual dexterity in patients with ASD (37), which hinders oral hygiene practice. Given the important role of salivary IgA in oral mucosal immunity, the decreased salivary IgA content in individuals with ASD found in this study may underlie the susceptibility of patients with ASD to oral pathogenic factors. We propose that restoring salivary IgA levels in ASD may prevent the development of various oral diseases.

In the submandibular glands of VPA mice, we observed a significant decrease in *Pigr* mRNA and protein levels compared with those in control mice. Indeed, the *Pigr* mRNA level was found to be positively correlated with the mouse salivary IgA concentration. Given the important role of PIGR in IgA transport, an impaired transportation of IgA into saliva is highly likely to cause a decrease in the mouse salivary IgA content. Other factors that may affect salivary IgA level were also examined; however, none were able to explain a reduction in the salivary IgA content. For instance, serum IgA level and the expression of genes that regulate salivary IgA were found to be significantly elevated in VPA mice.

The results of previous studies on ASD serum IgA levels have been variable. Some researchers have reported reduced levels of serum IgA in patients with ASDs (38, 39), while others have found no significant change (40). The heterogeneity of the results may be attributable to the different sample sources, sample sizes, and detection methods. Future studies should include larger sample sizes of patients with ASDs and individuals in animal models to determine whether they present any serum IgA alteration. In the present study, the expression of salivary IgA regulatory genes in the VPA mice submandibular glands, such as *Tnfsf13* and *Tnfsf13b*, was found to be upregulated. The tumor necrosis factor (TNF) family members TNFSF13 and TNFSF13b bind to receptors on B cells to promote IgA class switching (24), and may be responsible for the downstream upregulation of *Iga* and *Jchain* expression observed in this study. A similar tendency was observed only for another proinflammatory cytokine in the TNF family: TNFα. VPA rats were associated with a significant increase in the TNF-α protein level in the frontal cortex, cerebellum, hippocampus, and striatum (19), whereas in the duodenal and colonic tissues of children with ASD, a higher proportion of CD3+TNFα+ cells was detected (41). Consistent with the pro-inflammatory tendency of the central nervous system (42, 43), circulatory system (40, 44–46), and gastrointestinal tract (41, 45) in ASD, these findings provide new insights into the activated ASD immunophenotypes. However, the reduction in the salivary IgA and *Pigr* expression in the submandibular glands was incongruent with the internal state of activated oral mucosal immunity in the submandibular glands of VPA mice. This contradiction could be explained by the hypothesis that external environmental factors such as oral bacteria decrease salivary IgA transportation through PIGR modulation.

We confirmed the direct regulation of PIGR in HSG cells by *Streptococcus mutans* through *in vitro* experiments. A previous study reported similar results from *in vivo* experiments: salivary gland epithelial cells isolated from patients infected with oral *Streptococci* showed reduced *Pigr* expression (47). *Streptococcus mutans* is the major cause of dental caries (48); therefore, its inhibitory effect on *Pigr* is reasonable because it reduces the transportation of anti-caries IgA into the saliva. The role of other bacteria in *Pigr* regulation has been suggested (28), following the observation of increased ileal *Pigr* expression in germ-free mice colonized with *Bacteroides thetaiotaomicron* (49). *Bifidobacterium lactis* and *Escherichia coli Nissle* can also promote *Pigr* expression in human colonic epithelial cells (50, 51). Toll-like receptors (TLR) recognize bacterial components and transfer intracellular signals to activate the NF-κB and MAPK pathways, which mediate downstream pro-inflammatory responses. The specific mechanism by which *S. mutans* act on PIGR in HSG may involve the TLR/NF-κB/MAPK signaling pathway (52, 53). In line with Bruno's study, which reported that *Pigr* expression could be induced by *Escherichia coli Nissle* in human colonic epithelial cells via the TLR and NF-κB pathways (51), we demonstrated that *Streptococcus mutans* induced PIGR downregulation in HSG also involved the NF-κB pathway. It will be necessary to explore the role of TLR and the functionally relevant MAPK pathway further in future studies.

ASD remains behaviorally diagnosed based on DSM-5 criteria with no objective biomarker (54), which often leads to a delayed diagnosis since the autistic behaviors can only be identified after the full establishment of ASD. Thus, the identification of ASD biomarkers is required for more pre-symptomatic and comprehensive ASD diagnoses (55). Increasingly, research in this field is elucidating promising ASD biomarkers, such as blood amino acids (56), fecal microbiome (57), and fecal IgA (58). Qiao et al. proposed an oral microbial index for ASD diagnosis and achieved an accuracy of 96.3% (9). Another study also reported that oral bacteria, such as *Riemerella anatipestifer* and *Actinobacteria*, were correlated with restricted/repetitive behavior and social effect in children with ASD (8). The salivary IgA content is closely related to oral microbiota composition, which prompted us to evaluate whether salivary IgA could also serve to diagnose ASD. In this study, we found that human salivary IgA level has the potential to be applied in ASD diagnosis and the decreased salivary IgA content was significantly correlated with the severity of autistic-like behaviors in mice, indicating that salivary IgA is a potential biomarker for diagnosing ASD and evaluating the severity of autistic behaviors. Compared with blood biomarkers, fecal biomarkers, and oral microbiota, salivary IgA testing is relatively low-cost, and saliva is easier and less invasive to sample. Other immune components and microbial metabolites in saliva may also be combined with the salivary IgA for ASD diagnosis to optimize diagnosis accuracy in future studies (9).

## Limitations

First, ASD comprise a spectrum of heterogeneous diseases with a clinical presentation that is highly variable. Although this study performed both human and animal experiments, the sample sizes of the ASD and typically developing children were small, and the VPA-treated ASD mouse model could only represent a subset of ASD phenotypes. Enlarged human sample size and the inclusion of other genetic, environmental, and idiopathic mouse models of ASD are recommended for future studies. In addition, the male to female ratio of the TD children was 27:8, whereas that of the ASD children was 31:5. Although this gender bias was close to the male preponderance among patients with ASD (59), it may also affect the experimental outcome. Second, the severity scores of aberrant behaviors in the recruited ASD cohorts need to be taken into consideration in future studies to examine the relationship between human salivary IgA and autistic behaviors. Third, considering the complexity of the oral microbiota interaction, the modulation of epithelial *Pigr* cannot be simply attributed to a single bacterial genus. Thus, the effects of other bacteria with altered abundance in subjects with ASD need to be thoroughly investigated. Finally, this study mainly focused on the phenotype of oral mucosal immunity in ASD and the correlative relationship within and did not explain the causal effects of oral bacteria on ASD pathogenesis, which warrants further study.

## Conclusion

Despite these limitations, decreased salivary IgA levels in both patients with ASD and the VPA-treated ASD mouse model were observed in the present study. Furthermore, the diagnostic value of human salivary IgA level and a correlation between mouse salivary IgA content and mouse autistic-like behaviors were reported. The downregulation of *Pigr* in the submandibular glands of VPA mice was noted in the *in vivo* experiments, which was positively correlated with the reduced salivary IgA in VPA mice. *In vitro* experiments demonstrated that ASD-related *Streptococci*, whose abundance was increased in ASD oral microbiota, could modulate the downregulation of PIGR in HSG cells. We observed decreased salivary IgA content in individuals with ASD and proposed the modulation of the salivary gland *Pigr* by ASD-related oral microbiota. This study provides novel insights into the diagnosis and oral health care of individuals with ASD and sheds light on the mucosal immunophenotype of ASD in the oral cavity.

## Data Availability Statement

The original contributions generated for the study are included in the article/Supplementary Material, further inquiries can be directed to the corresponding author/s.

## Ethics Statement

The studies involving human participants were reviewed and approved by Ethics Committee of the School and Hospital of Stomatology, Tongji University. Written informed consent to participate in this study was provided by the participants' legal guardian/next of kin. The animal study was reviewed and approved by Ethics Committee of the School and Hospital of Stomatology, Tongji University.

## Author Contributions

MW, XM, and YL designed the study. WG, YQ, BL, XZ, and RX collected the human saliva samples. WG conducted the experiments and wrote the manuscript. XZ, RX, BL, YQ, MW, XM, and YL revised the manuscript. All authors have read and approved the manuscript.

## Conflict of Interest

The authors declare that the research was conducted in the absence of any commercial or financial relationships that could be construed as a potential conflict of interest.
