# Supplementary material for: The Alteration of Salivary Immunoglobulin A in Autism Spectrum Disorders
Source: Front Psychiatry. 2021 May 21;12:669193. doi: 10.3389/fpsyt.2021.669193 (PMC8175640; doi:10.3389/fpsyt.2021.669193)
Supplement: Supplementary file 1 [file Data_Sheet_1.docx]

Supplementary Material

**Supplementary** **Table 1. Primer sequences.**

| Gene | Primer sequence |
| --- | --- |
| *Gapdh* | 5′-AGGTCGGTGTGAACGGATTTG-3′  5′-TGTAGACCATGTAGTTGAGGTCA-3′ |
| *Aid* | 5′-CCAGGAACCGCTACTCGTTT-3′  5′-GGTCCGTCTCAGGCACTATG-3′ |
| *Tnfsf13* | 5′-TCACAATGGGTCAGGTGGTATC-3′  5′-TGTAAATGAAAGACACCTGCACTGT-3′ |
| *Tnfsf13b* | 5′-TGCTATGGGTCATGTCATCCA-3′  5′-GGCAGTGTTTTGGGCATATTC-3′ |
| *Iga* | 5′-CCTAGTGTTTGAGCCCCTAA-3′  5′-GGAAGTGCAGGGATACTTTG-3′ |
| *Jchain* | 5′-ACGACGAAGCGACCATTCTT-3′  5′-ATCCTCGGTGGAAGGGATGA-3′ |
| *Tgfβ1* | 5′-GCAACATGTGGAACTCTACCAGA-3′  5′-GACGTCAAAAGACAGCCACTCA-3′ |
| *Aldh1a1* | 5′-CTCCTCTCACGGCTCTTCA-3′  5′-AATGTTTACCACGCCAGGAG-3′ |
| *Aldh1a2* | 5′-GACTTGTAGCAGCTGTCTTCACT-3′  5′-TCACCCATTTCTCTCCCATTTCC-3′ |
| *Pigr* | 5′-GTTCCTGAGTTGCCGAGTGA-3′  5′-TCTGGATCCCTCTTCCCCAG-3′ |

**Supplementary Figure 1.** The growth curve of *Streptococcus mutans* (ATCC 25175). A single colony of *Streptococcus mutans* (ATCC 25175) was inoculated into 10 ml brain heart infusion broth, and the optical density was measured at different time points.
